# Vertical growth pattern as a determinant of mandibular asymmetry

**DOI:** 10.12669/pjms.38.5.5294

**Published:** 2022

**Authors:** Maria Habib, Tabassum Ahsan, Omair Majeed, Faisal Faheem

**Affiliations:** 1 Maria Habib, Assistant Professor, Department of Orthodontics, Bahria University Medical and Dental College, Karachi, Pakistan; 2 Tabassum Ahsan, Associate Professor, Head Department of Orthodontics, Bahria University Medical and Dental College, Karachi, Pakistan; 3 Omair Majeed, Senior Registrar, Department of Orthodontics, Bahria University Medical and Dental College, Karachi, Pakistan; 4 Faisal Faheem, Senior Lecturer, Researcher, Department of Physical Therapy, Bahria University Medical and Dental College, Karachi, Pakistan

**Keywords:** Asymmetry, Vertical dimention, Mandibular asymmetry, Habets technique

## Abstract

**Objectives:**

To determine normal mandibular linear values in three vertical groups, to compare right and left side to highlight a prevalent pattern towards mandibular asymettry.

**Methods:**

This is a descriptive cross sectional study in which pre-orthodontic treatment panoramic radiographs and lateral cephalograms of 224 patients (between 18 to 34 years) undergoing treatment in Orthodontic Department of Bahria University Medical and Dental College were used and traced on an acetate sheet. There were 74 patients in high angle,76 and 78 in low angle and normal angle group respectively based on their vertical growth pattern using SNMP angle. Condylar height (CH) and Ramal height (RH), and condylar plus ramal height (CH+RH) measurements were done as previously described by Habets.

**Results:**

There was no statistically significant difference between the three groups as determined by one way ANOVA. Condylar height and Gonial angle showed statistically significant difference when right and left side was compared, with increased values on the right side.

**Conclusion:**

There was no statistically significant difference in condylar, ramal, and total asymmetry index between different vertical groups. Between the right and left sides, condylar height and gonial angles were significantly increased on the right side. However, Ramal height showed no significant difference. Ramal index has a strong linear correlation with total asymmetry index.

## INTRODUCTION

Symmetry is a theoretically ideal concept and it is now widely accepted from recent and classical texts^1^ that even those faces that are considered pleasing, do end up showing some degree of asymmetry. Therefore asymmetry is considered and classified only when there is a substantial difference between the two sides of the face that is quantifiable by a clinician.^2^ However due to the subjectivity of the facial esthetics, the threshold for clinical significance is not easily determined and therefore is dependent on the area of asymmetry, patient’s perception of the mis-proportion, clinician’s sense of balance, or the acceptable cultural norms of a particular ethnic group.^2^ Wolford proposed a few etiologies for non-pathological asymmetry that include genetics, intra uterine molding, natural growth variation and environmental factors.^3^

As far as growth and development are concerned, mandible is considered a separate entity in the craniofacial complex.^4^ Condyle being a growth center and having the highest growth potential in mandible, has the most effect on its growth pattern. Therefore, the shape and volume of condyle is a significant diagnostic variable of mandibular asymmetry. Studies have shown that age, gender, TMJ anatomy and occlusal forces, influence the morphology of condyle and therefore indirectly its growth.^5^ Ramus’ height also has an indirect contribution to mandibular asymmetry.^6^ Vertical growth pattern is determined by factors that are grossly divided into skeletal and dento-alveolar. There are three basic types of skeletal vertical growth patterns hyperdivergent, hypodivergent and normodivergent. SN-MP, FH-MP and MMA values on cephalomteric analysis are generally used to categorize the patients into these patterns.

Studies haven’t shown statistically significant variation of mandibular asymmetry between genders,^7^ yet variability has been found when comparing in vertical and sagittal malocclusions.^7^ Various indexes have been routinely used to find vertical mandibular asymmetry based on panoramic radiographs, cephalograms (lateral and posterior-anterior), Computed Tomograms (CT) and Cone Beam Computed Tomography (CBCT) analysis.^8^-^10^

Panoramic radiograph has been widely used for diagnosing mandibular asymmetry.^11^,^12^ Habet et al^13^ gave a method to evaluate mandibular vertical asymmetry by measuring right and left condylar and ramal heights on panoramic radiographs. Later this method was used to asses mandibular asymmetry in temporomandibular joint disorders^14^, posterior cross bites^15^, sagittal malocclusions^7^, unilateral and bilateral molar extractions^16^ and cleft lip and palate cases.^17^

Since panoramic radiograph is conveniently available for orthodontic patients and it has a sound validity in diagnosing mandibular asymmetry, the present study used this for finding the correlation between vertical pattern and mandibular asymmetry. A significant association in this regard might render CBCT analysis, solely for this purpose, an unnecessary radiation exposure. CBCT has been used in many of the previous studies mentioned above, but in third world countries where there is insufficient access to these modalities our study will benefit in not only the diagnosis of the asymmetry but also in identifying what might be normal for our population.

## METHODS

This study had a descriptive cross-sectional design. Pre-orthodontic treatment panoramic radiographs and lateral cephalograms of 224 patients (between 18 to 34 years) undergoing orthodontic treatment in the department of Bahria University Medical and Dental College were used for sample collection. Informed consent regarding future use of records for study purpose was taken at the time of beginning treatment. There were 74 patients in high angle, 76 and 78 in low angle and normal angle group respectively based on their vertical growth pattern using SNMP angle (high angle ≥38º, Low angle ≤26º, normal angle group 26-38º).^8^,^9^

### Sample size calculation:

Sample size was determined by using software G Power version 3.1.9.2 for windows. For calculating sample size for mandibular asymmetry, vertical growth patterns was classified into three groups. We used F-test repeated measures ANOVA with 95% confidence interval and 5% margin of error. The required sample was found to be 189 in total and 63 in each group.

### Exclusion criteria:

Patients with a history of orthodontic treatment, having TMJ disorders or any craniofacial syndrome were excluded from the study.A single digital machine by Durr Dental (Model type -Vista Pano, Model No DG-07C11T2, Power input 200-240 v, 50-60 Hz, 2.2KVa Max.) was used to take all OPGs Radiographs were done by a technician who followed the standard patient positioning protocols. The tracings were done by a single calibrated examiner on an acetate sheet for all the patients, using a digital caliper with 0.1mm sensitivity. The readings were taken for both the right and left sides. A line connecting the most lateral point of the condyle (O1) and the ascending ramus (O2) was drawn and mentioned as ‘A’. Ramus height was the distance between the points O1 and O2, and called ‘RH’. Line ‘B’ was drawn perpendicular to line ‘A’, touching the most superior part of the condyle. The vertical distance from this line till O1 was measured and named the condylar height, ‘CH’. To reduce intra operator bias, 5 cases were randomly retraced to calculate the readings, the Kappa Statistics was found to be 0.67 indicating good agreement. Gonial angle (G.A) was taken between line A and tangent to lower border.

A separate examiner did the asymmetry analysis who was unaware of the patients growth pattern to ensure blindness. Condylar height(CH) and Ramal height (RH), and condylar plus ramal height (CH+RH) measurements were done as previously described by Habets et al,^13^ and widely used as Habets method. The vertical indexes were calculated using the following formula:


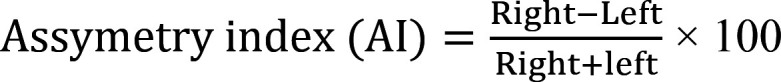


**Fig.1:**
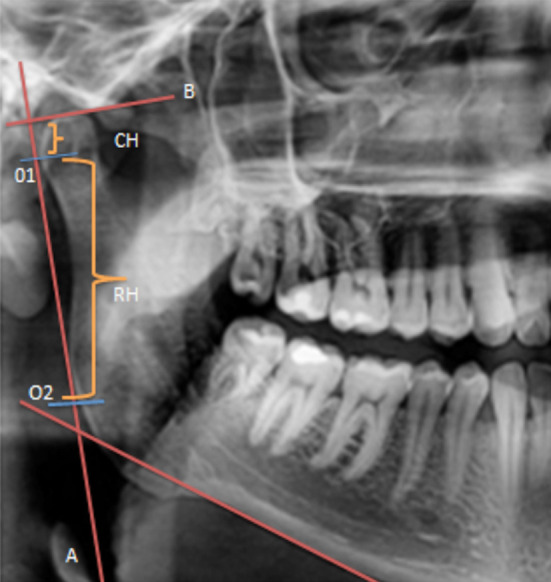
Showing the variables measured on the OPG.

Based on the AI value for each patient the results were classified into four categories of asymmetry: not significant (NS) asymmetry, when AI was between 0 and 2.99%; light (L), when AI was between 3 and 5 %; moderate (M), when the index was greater than 5 %, but less than or equal to 10 %; and severe (S), when AI was more than 10 %.^18^

### Statistical analysis:

SPSS version 23 software package of windows was used with p<0.05 considered statistically significant. Shapiro-Wilks test was used to ensure normal distribution. Intra examiner reliability calculated using kappa analysis was moderate (0.67).One way ANOVA was used to compare indexes CAI, RAI, CRAI between vertical groups (high, low and normal angle).

A student t-test was used to compare right and left linear (CH, RH, CRH) and angular (G.A) measurement of mandible to find out a prevalent pattern of asymmetry. Pearson-correlation was used to find out the effect of condylar index and ramal index on total asymmetry index.

### Ethical approval:

Ethical approval was taken from the Ethical Review Committee of Bahria University Medical and Dental College (ERC 27/2021).

## RESULTS

Our sample had a total of 228 patients, having a mean condylar asymmetry index value of 4.9% and ramus asymmetry index of 0.04%. The mean values of the condylar and ramal asymmetry index for high angle, low angle and normal angle cases is shown in Table-I. The values indicated that the low angle group showed moderate severity while the high and normal angle groups showed light asymmetry index. The ramal index and total(C+R) index show no significant asymmetry according to the criteria given by Ramirez at al.^18^

**Table I:** One-Way Anova to compare indexes between groups with significant p value>0.05.

	High Angle (n= 74)	Low Angle (n=76)	Normal Angle (n=78)	p- value

	Mean ±SD			
Condylar Assymetry Index (CAI)	3.77 ± 7.41	6.22 ± 6.54	4.8 ± 2.6	*0.62*
Ramal Assymetry Index (RAI)	0.2 ± 5.86	-0.39 ± 4.61	0.06 ± 3.7	*0.72*
Condylar and Ramal Assymetry Index (CRAI)	0.62 ± 4.9	0.21 ± 3.5	0.63 ± 3.11	*0.75*

Gonial angle represented the vertical growth pattern in its classic form, hight gonial angle in high angle cases and vice versa.There was no statistically significant difference between the three groups as determined by one way ANOVA (Table-I). Condylar height and gonial angle showed statistically significant difference when right and left side was compared, with increased values on the right side (Table-II).

**Table II:** Paired sample t test between right and left sides with significant p value>0.05.

	Right n=228	Left n=228	p-value

	Mean ±SD		
Condylar and Ramal Height (CRH)	52.4 ± 5.9	52.0 ± 6.4	0.20
Condylar Height (CH)	8.14 ± 2.4	7.25 ± 2.3	0.00*
Ramal Height (RH)	44.3 ± 5.6	47.2 ± 5.9	0.93
Gonial Angle (GA)	124.6 ± 8.2	123.6 ± 7.7	0.01*

**Fig.2:**
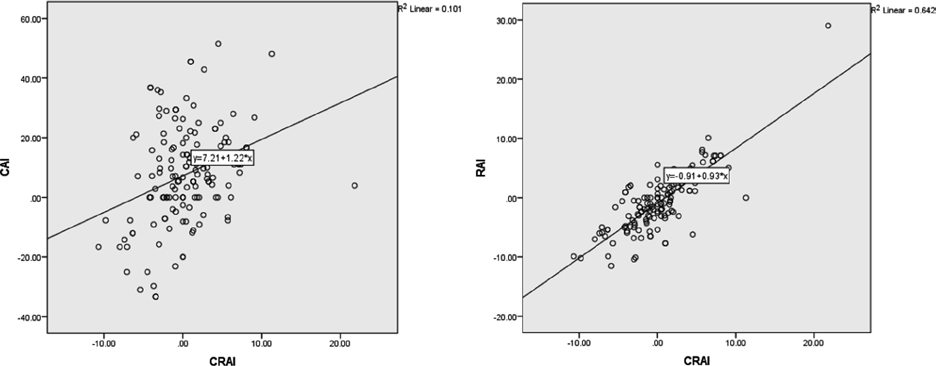
Scatter graph showing strength of correlation between RAI and CRAI.

Pearson correlation showed a positive linear correlation of both condylar and ramal asymmetry index with total asymmetry index. Ramal index being more strongly correlated than condylar index as shown by scattergraph (Fig.2).

## DISCUSSION

Panoramic radiograph was used in this study to reinforce maximum utilization of routinely available radiographs and also for providing bilateral information. This study generates linear measurement of right and left sides of condyle and ramus, and calculates an index between them according to Habets.^12^ Another index Kjellbergs is also used using the same measurements but slightly different formula. CH is more precisely measured in Kjellbergs taken from the head of the condyle to the depth of the mandibular notch, when compared with Habets.^11^ However simplicity of measurement and analyses was the reason Habets was used in this study .

Out of the 228 cases in our study, 35(15%) cases had CRAI more than 3% showing asymmetry. The original Habets^13^ study showed a 15 out of 92(16%) cases having more than 3% CRAI. Our results did not show a statistically significant difference between asymmetry indexes of the three vertical groups against the assumption that high angle cases are more associated with asymmetry due to their pronounced gonial angles and more growth at the condyle.^19^,^20^ This result was however in agreement with Sofyanti et al who conducted a similar study using OPGs.^5^ He also found no statistically significant difference in asymmetry based on vertical patterns. To avoid magnification and distortion error associated with OPG, recent studies use CBCT and 3D imaging which also showed no relevance of vertical pattern on mandibular asymmetry.^3^ A study on linear and volumetric asymmetries in adults with different skeletal and vertical classes by Mendoza lV et al.^20^ also failed to show any association between presence of asymmetry and vertical pattern.

Our results showed a statistically significant difference in the condylar heights between right and left sides. Mendoza IV et al.^20^ did not find any statistical difference between condylar and ramal heights on right and left sides. This can be explained by the sample selection where they performed the study on caucations, however magnification error in OPG could also be the reason for variation in results.

Ming talk chew^21^ analyzed the extent and management of dento-facial deformity in multi ethnic Asian populations, 91.5% of the sample showing marked asymmetry was Chineses and 68% had class 3 malocclusion, explaining the non-significant asymmetry values of our sample. Haraguchi et al also found that in a study sample of 220 Class III Japanese patients, 56% had soft tissue asymmetry and 80% had some degree of hard tissue asymmetry.^22^

Prevalence and severity of mandibular asymmetry in Caucasian adults was also studied by Shane J. McCrea and Mark Troy by comparing right and left side using Habets analysis. They concluded that right mandibular ramus was longer in both genders^23^, same result was also shown by Skvarilová et al.^24^ Both these studies were in accordance to ours showing asymmetry dominance towards the right side. Kaur M et al.^25^ evaluated gonial angle on OPG and lateral cephalogram and found increased gonial angle values on the right side (123.1) than the left (122.5) showing the same pattern . However, the difference in right and left side can be entirely subjected to patient positioning error.

Pearson correlation results shows ramal linear measurement being decisive in expression of asymmetry in the form of positive linear correlation, as also shown by Chen Fang et al.^26^ According to Leung LY mandibular ramal asymmetry contributes largely to mandibular asymmetry along with mandibular body and chin point and lastly condylar growth. Condylar height values increased on the right side support the philosophy of dominant side which is not supported by evidence. Kelesl P et al studied facial asymmetry in right and left handed adults through posterio anterior cephalograms and saw an opposite pattern, this could be because in this study condyle was not considered as a separate entity.^27^ Asymmetry can present in a varied fashion and vertical dimention is clearly not a decisive factor in this regard. However, this study gives us the norms for various linear and angular mandibular measurements for our population which can be diagnostically significant.

### Limitations of the study:

Although three dimensional studies are gold standard in the diagnosis of asymmetry, this study used two-dimensional imaging.Right and left side dimensional changes can be subjected to positional errors while Habets method is tedious and manually cumbersome.

## CONCLUSION

There was no statistically significant difference in condylar, ramal, and total asymmetry index between different vertical groups.Between the right and left sides, condylar height and gonial angles were significantly increased on the right side. However, Ramal height showed no significant difference.Ramal index has a strong linear correlation with total asymmetry index.

### Authors’ Contribution:

**MH,** Proposal/project development /manuscript writing/data collection.

**TA,** Manuscript writing/editing.

**UM,** Assisted in Data collection.

**FF,** Data Analysis.
